# Quantity and/or Quality? The Importance of Publishing Many Papers

**DOI:** 10.1371/journal.pone.0166149

**Published:** 2016-11-21

**Authors:** Ulf Sandström, Peter van den Besselaar

**Affiliations:** 1 KTH Royal Institute of Technology, INDEK, SE-100 44 Stockholm, Sweden; 2 VU University Amsterdam, Network Institute & Institute for Social Resilience Amsterdam, Amsterdam, The Netherlands; Universidad de las Palmas de Gran Canaria, SPAIN

## Abstract

Do highly productive researchers have significantly higher probability to produce top cited papers? Or do high productive researchers mainly produce a sea of irrelevant papers—in other words do we find a diminishing marginal result from productivity? The answer on these questions is important, as it may help to answer the question of whether the increased competition and increased use of indicators for research evaluation and accountability focus has perverse effects or not. We use a Swedish author disambiguated dataset consisting of 48.000 researchers and their WoS-publications during the period of 2008–2011 with citations until 2014 to investigate the relation between productivity and production of highly cited papers. As the analysis shows, quantity does make a difference.

## Introduction

One astonishing feature of the scientific enterprise is the role of a few extremely prolific researchers [1]. Thomson Reuters gather them in the *Highly Cited Researchers* database, where they are listed and recognized per area. Using another database, *Scopus*, Klavans & Boyack phrase them ‘superstars’ in a large-scale study of publication behaviour, thereby showing that superstars publishes less in isolated areas (retrieved using a clustering procedure), in dying areas, or in areas without an inherent dynamics [2]. Highly productive and cited researchers tend to look for the new opportunities. Obviously, the highly productive researchers have to be taken into consideration for many reasons, both for science policy and for scholarly understanding of how the science system works.

Within bibliometrics there is a discussion on how to identify and measure “superstars”, and many papers discuss the correlation between the various indicators developed for performance measurement. One of the stable outcomes is that there is a high correlation between the numbers of papers a researcher has published and the number of citations received [3]. From that perspective, both indicators tend to measure the same attribute of researchers, as is actually materialized in the introduction of the H-index [4]. The progress of science rests on the huge amount of effort and publications, but the number of real discoveries and path breaking new ideas is rather small. This has led to a different focus, and the discussion about impact has shifted from counting numbers of citations to more qualified types of citations and weighted publications. Instead of counting publications and citations, the decisive difference in this perspective is whether a researcher contributes to the small set of very high-cited papers. Different thresholds are deployed, from the top 1% highly cited papers to the top 10% highly cited papers or with the CCS method proposed by Glänzel & Schubert [5]. Generally speaking, only when a paper reaches such a citation level, it contains a distinctive result that contributes to scientific progress. Increasingly, performance measures take this selectivity into account, and when calculating overall productivity and impact figures for researchers, papers (productivity) and citations (impact) are weighted differently depending on the impact percentile the paper belongs to [6].

If one agrees that in science it is all about top (cited) publications, the question comes up what an efficient publication strategy would look like. Is publishing a lot the best way–or does that generally lead to *normal science*, Kuhn [7], with only low impact papers? The total number of citations received may still be large, but no top papers may have been produced. This was already the core of Butler’s critique on the Australian funding system [8] and is also the underlying idea of emerging movements in favour of ‘slow science’ like e.g. in the Netherlands; there the ‘science in transition’ movement [9] was able to convince the big academic institutions to remove productivity as a criterion from the guidelines for the national research assessment (SEP). The underlying idea is that quality and not quantity should dominate–and that with all the emphasis on numbers of publications, the system has become corrupted, see the discussion in The Leiden Manifesto (Hicks et al. [10]), and the Metric Tide report (Wilsdon et al. [11]).

However, others seem to see this differently. Firstly, recent empirical research suggests that on the long run, average output per researcher (corrected for the number of co-authors) has not increased at all [12]. This suggests that much of the debate on output indicators and output funding leading to perverse effects may be wrongly directed. Secondly, in his work on scientific creativity, Simonton has extensively argued that (i) having a breakthrough idea is a low probability event that happens by chance, and therefore that (ii) the more often one tries, the higher the probability to have a ‘hit’ so now and then [13]. Also other contextual factors influence the chance for important results, but overall, the number of tries (publications) is the decisive variable. This would also explain why Nobel laureates often have many more publications than normal researchers. Zuckerman states that laureates publish at a much higher rate [14], and Sandström shows the early recognition of papers by later Nobel laureates [15]. The more often a researcher tries out an idea (and publishes it) the higher the probability that there is something very new and relevant, and atypical for the scientific community [16].

**Fig 1:**
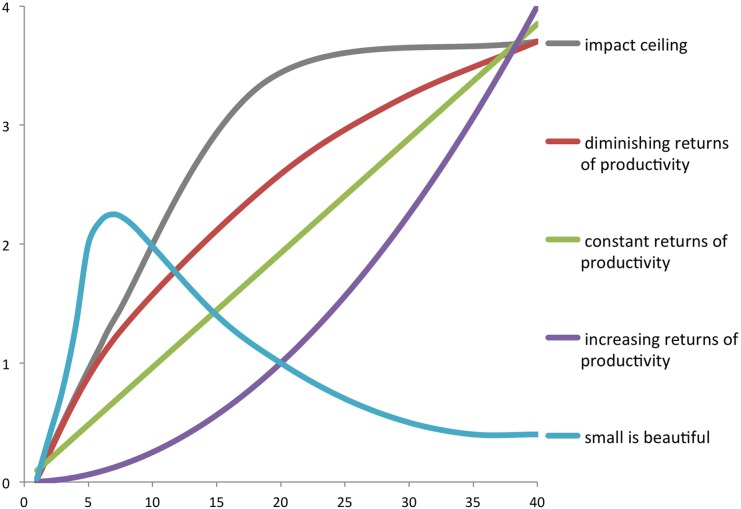
Five types of possible relationships between the number of top papers and all papers.

This brings us to the question what the relation is between overall output (number of publications) and the number of high impact papers of researchers. Several possibilities exist, and only the last two would be evidence that more is not better:

- Increasing returns from productivity: The number of top papers increases faster than the total output (the purple line in Fig 1). So, the higher the total output of a researcher, the more he/she contributes to scientific progress in an absolute (number of top papers) and in a relative sense (share of top papers).
- Constant returns from productivity: The number of top papers increases proportionally with total output (the green line in Fig 1). So the higher the total output of a researcher, the more he/she contributes to scientific progress in an absolute sense, but in a relative sense there is no difference.
- Positive but declining returns from productivity: The number of top papers increases but less fast as total output (the red line in Fig 1). So the higher the total output of a researcher, the more he/she contributes to scientific progress in an absolute sense. However within the corpus the share of lower impact papers increases.
- A limit to impact: It might be the case that for every researcher there is a limit to the number of good ideas that can be produced. Above some productivity threshold, papers will merely repeat already published ideas, and will not contain new or good ideas anymore. If that would be the case, above that productivity threshold the number of top cited papers remains about constant (the grey line in Fig 1).
- Small is beautiful: Publishing a lot is detrimental to quality. Those who write a lot may have even less top papers than those that are more selective: small is beautiful, represented by the blue line in Fig 1.

So the focus of this paper is on the researcher and his/her oeuvre: does higher productivity result in a higher absolute and/or relative number of top cited papers? And the unit of analysis is not the paper: that is because a paper generally has more authors, who individually can be more or less productive. The answer to our question may inform our understanding of knowledge production and scientific creativity, but is also practically relevant for selection processes, and as explained above for research evaluation procedures: is high productivity a good thing, or a perverse effect and detrimental to the progress of science as it does not add to our knowledge, but only to a waste of paper?

Not much research on this issue is available. Abramo et al. [17] tried to answer the question by use of an Italian database of unique authors. They find only a moderate correlation between productive scientist and authors of highly cited articles. One obvious problem with their article is that they take for granted that top scientists work in solitude or with each other, but never work with younger and less experienced researchers. We will discuss that problem although we are not able to solve the problem in full in this paper.

Ioannidis et al. [18] showed that less than 1% of all researchers that published something (indexed in Scopus) between 1996 and 2011 did publish in each of these 16 years, and that this small set of core scientists are far more cited than others. They account for 41.7% of all papers in the period under study and 87.1% of all papers with >1000 citations. Also Kwiek shows that a large share of total publications comes from a relatively small group of high productive researchers, but does not go into the issue of quality of the papers [19]. Larivière & Costas do focus on top publications and they report stronger findings: “especially for older researchers (……) who have had a long period of time to accumulate scientific capital, there can never be too many papers.” [20]

### Data and Methods

In order to answer our question, we use the 74.000 WoS-publications 2008–2011—with a citation window until 2014—of all researchers with a Swedish address using the following document types in databases SCI-E, SSCI and A&HCI: articles, letters, proceeding papers and reviews.

For identifying authors and keeping them separate we use a combination of automatic and manual *disambiguation* methods. An algorithm for disambiguating unique individuals was developed by Sandström & Sandström [21], and further developed by Gurney et al. [22]. It proceeds fast, although requires supplementary manual cleaning methods. The deployed method takes into account surnames and first-name initials, the words that occur in article headings, and the journals, addresses, references and journal categories used by each researcher. There is also weighting for the normal publication frequency of the various fields [21; 23].

As indicated the data covers 74.000 articles and 195.000 author shares that have been judged to belong to Swedish universities or other Swedish organisations. In a few cases, articles from people who have worked both in Sweden and in one or more Nordic countries have been kept together, and articles have thus been included even if they came into being outside Sweden (the process of distinguishing names is thus carried out at Nordic level). This was done as the academic labour market to quite a large extent is Scandinavian.

All articles by each researcher are ranked, based on received citations and according to the about 260 subject categories as specified in the Web of Science, and the articles are divided into CSS (Characteristic Scores and Scales) classes (0, 1, 2, 3). While measures based on percentile groups (e.g. PPtop1% etc.) are arbitrarily constructed, CSS have some advantages concerning the identification of outstanding citation rates [5]. The CSS method is a procedure for truncating a sample (e.g. a subfield citation distribution) according to mean values from the low-end up to the high-end. Every category of the CSS created using this procedure helps to identify papers that fulfil the requirements for being cited above the respective thresholds. In this paper we will use two levels, level CSS1 and CSS3, which in the former case cover the 37% most cited papers, and in the latter case the about 3.5% of most cited papers: the “outstandingly cited papers” [24].

In the following we will investigate the relation between quality and quantity in several different ways. We do this, as from a methodological perspective different options are open, without a convincing argument which one would be the better. By using a variety of methods, we avoid to produce results that are only artefacts of a specific method.

1. We investigate whether the *number of CSS3* papers increases with productivity (purple, green, red line in Fig 1), and whether the *share of CSS3* papers as part of all papers increases with productivity (the purple line in Fig 1). A main issue is whether there is a CSS3 ceiling (the grey line in Fig 1), or whether high productive authors actually have a low impact, and whether small is beautiful (the blue line). We first calculate the average number of CSS3 papers, given the productivity level of authors. In order to do so, we classify all about 48.000 Swedish researchers into productivity classes: productivity class 1 has one publication in the four years period under study, class 2 has two, class 3 has three to four, class 4 has five to eight, class 5 has nine to sixteen, class 6 has seventeen to 31 publications, and finally class 7 covers researchers with 32 or more publications. The classification bins have been sized to be roughly even on a logarithmic scale. For each of these classes, we calculate the average number of papers in the CSS3 class. This is done for the whole set, and for the eight distinguished research fields separately (Table 1) based on a classification developed by Zhang et al. [25] (the field of humanities added to the classification by us). Publications are integer counted, and citations are field normalized. The *average number of papers in CSS 3* is not an integer, as on average only one out of some 30 papers belongs to CSS3.
2. We do a regression with the total number of integer counted (IC) publications of an author as the independent variable, and the also integer counted number of top cited publications of the same author. We do this for each of the definitions of ‘top cited’ that were discussed above, using field-normalized citations (using normalization to subject category, publication year and document category). This is done for the total population of (publishing) researchers, without normalizing for field-based productivity differences. As the total set of researchers is dominated by life and medical sciences and by natural sciences, and as these groups have comparable average publications and citations, we assume that this does not influence the results. However, under point (iv) below, we introduce a way of taking field differences in productivity into account.
3. We do the same analysis as under (ii), but use fractional instead of integer counting of publications and top-cited publications. This helps to investigate the effect of different ways of counting on the relations under study.
4. We introduce field-normalized (fractional counted) productivity, and calculate the relation between in this way defined productivity and having at the number of fractionally counted publication in CSS1 and CSS3. As we produced field normalized output counts, we can provide an integrated analysis of all researchers across all fields. This is done with a method–Field Adjusted Production (FAP)—based on Waring estimations. This method was initially developed by Glänzel and his colleagues [26, 27], during the 1980s, and further explained and tested in Sandström & Sandström [21], see also [23]. Basically, the method is used in order to compensate for differences in ‘the normal rate of scholarly production’ between research areas. To this end, all journals in the Web of Science have been classified according to five categories (applied sciences, natural sciences, health sciences, economic & social sciences, and art & humanities). Note this is a different classification than the one used above. This is because for productivity, the distinction between basic and fundamental should be more explicitly taken into account. We collapse some fields, but split everywhere applied research from basic. This categorisation of journals into macro fields is based on Science Metrix classification of research (<http://science-metrix.com/en/classification>).

**Table 1:** Number of top cited papers (CSS3) by productivity class* and field.

Publ	Agriculture & Food Science	Biology, Environ & Geography	Life & Medical Science	Chemistry Physics & Engineering	Computer Sci & Math	Humani-ties	Psychology Education	Sociology Economics& Pol Sci
1	0.03	0.03	0.02	0.03	0.00	0.01	0.03	0.02
2	0.08	0.08	0.06	0.06	0.01	0.01	0.06	0.05
3–4	0.15	0.15	0.10	0.07	0.05	0.19	0.07	0.11
5–8	0.26	0.30	0.20	0.16	0.14	0.17	0.19	0.26
9–16	0.50	0.66	0.51	0.32	0.14		0.69	0.56
17–32	1.24	1.47	1.07	0.71			2.16	0.81
>32			3.51	3.05				

If productivity classes contain less than 0.5% of the papers, it was combined with the next lower class.

That explains why several cells are empty.

## Results

### (i) Does the probability of high-cited papers increase with productivity?

We calculated for each of the seven productivity classes the average number of CSS3 papers (Table 1). So, in productivity class 1 of Agricultural and Food science, researchers have published 1 paper in the period considered, and on average they have 0.03 paper in CSS3. In productivity class 2 of Agricultural and Food Science, authors have 2 papers and on average 0.08 papers in CSS3. Where the highest productivity classes are too small, they were combined with the next lower level. Inspecting Table 1 shows that for all fields the CSS3 score increases with productivity–only for the Humanities we found that the score in the highest productivity class 4 is slightly lower than in the productivity class 3, and for computer science & mathematics it is equal. So in general, the higher the productivity of a researcher, the more top-cited papers a researcher has.

**Fig 2:**
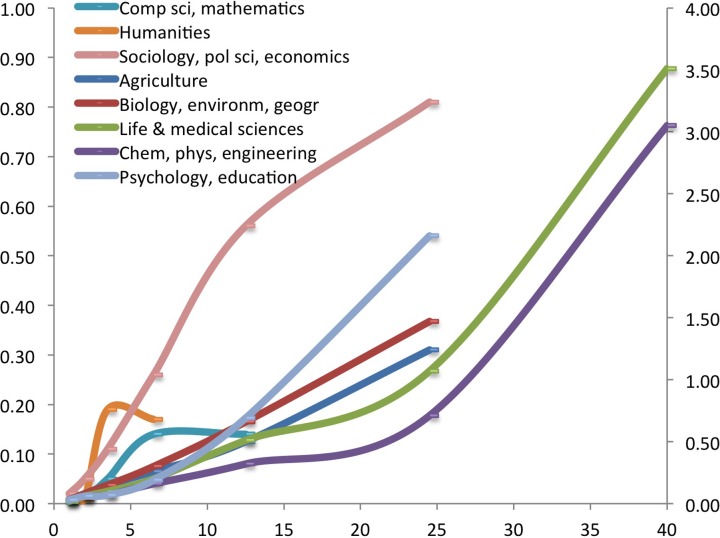
Average number of top cited papers (CSS3) by field and productivity class.

In Fig 2 we show the relation between productivity and authoring top cited papers (from Table 1) graphically, which makes it easier to compare the found patterns with the model of Fig 1. Fig 2 suggests increasing returns from productivity for the Sciences & engineering, the Life sciences, and for Psychology. A constant return from productivity is found for Environmental sciences & biology as well as for Agricultural science related fields. The Social sciences show decreasing returns, and finally, Computer science & mathematics and the Humanities show a ceiling for top cited papers. For the latter fields it should be noted that the output we take here into account (journal articles) may not be representative enough. Furthermore, these disciplines also have characteristics that may be relevant here [Whitley 28]. For example, fields like social sciences, humanities, and computer science have different audiences than only peers: the general public, professionals, policy makers etc. If especially the stars do publish often for those non-peer audiences, their total output increases, but this may not result in additional citations.

**Fig 3:**
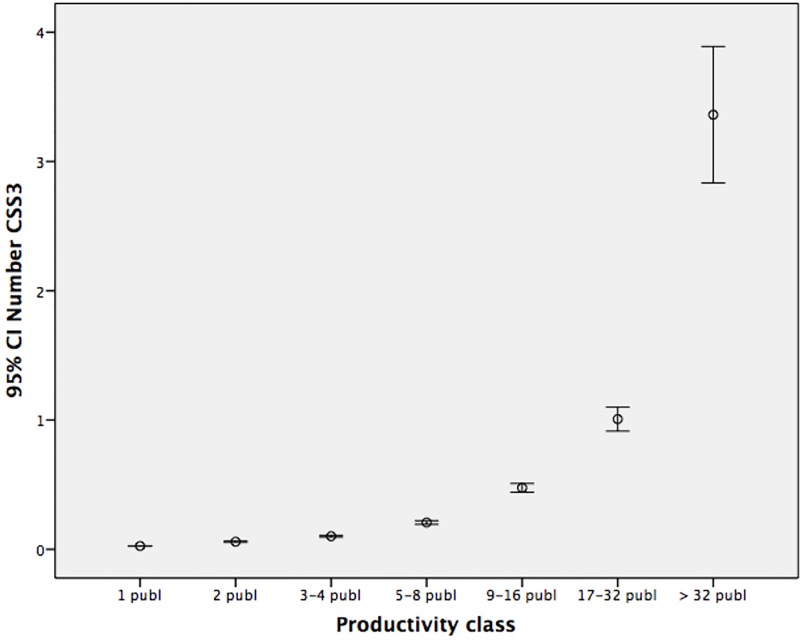
Means and 95% confidence intervals: number CCS3 by productivity class.

**Fig 4:**
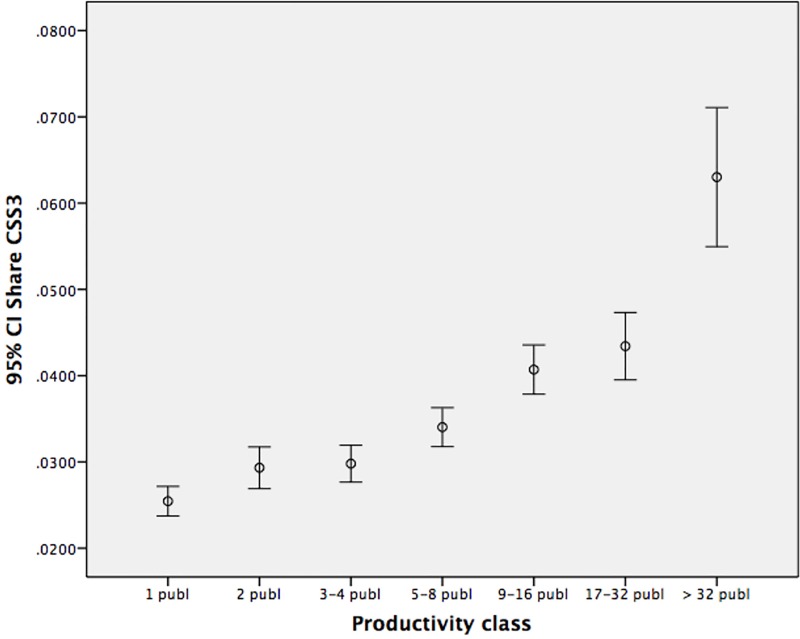
Means and 95% confidence intervals: share CCS3 by productivity class.

Are the differences between the productivity classes statistically significant? Anova tests show that the means of the *number* of CSS3 publications differ between the classes for all fields (Fig 3). Anova test for means of the *share* of CSS3 publications does not result in significant differences between class 2 and class 3, and between class 5 and class 6 (Fig 4). But over the whole range, also the *share* of top cited papers increases with output. So, more seems indeed better. Data are rather skewed and the Levene Test indicates no homogeneity of variance, therefore non-parametric tests were used as well. We tested whether the medians of the different productivity classes are significantly different, and use the Kruskal-Wallis test of the mean rank differences of the number and share of CSS3 papers between the productivity classes. This leads to the same findings as the Anova’s.

Repeating the same analysis for the individual fields gives similar outcomes for means and medians, although the differences are (because of the smaller number of cases in each of the fields) more often non-significant. The estimated means of the number and share of CSS3 papers for the individual fields, as well as the 95% confidence levels are in Supporting information. The main ‘deviant’ patterns are for computer science, for the humanities and for the social sciences, where the highest productivity classes actually have a lower share of CSS3 papers than the one but highest class–as was also visible in Fig 2.

### (ii) The effect of productivity on the number of high-cited papers

**Fig 5:**
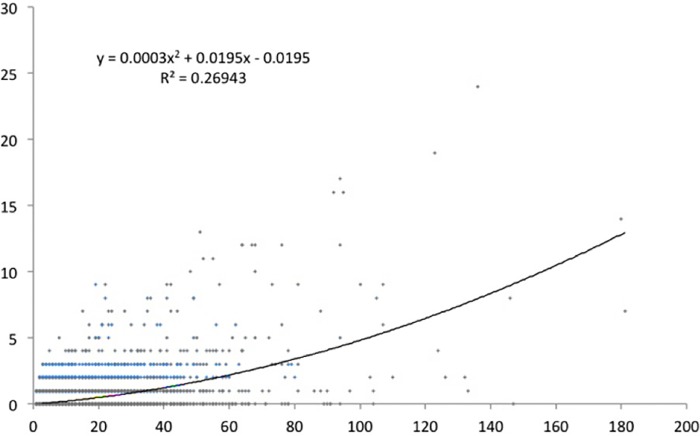
Top1% cited papers by total number of papers.

**Fig 6:**
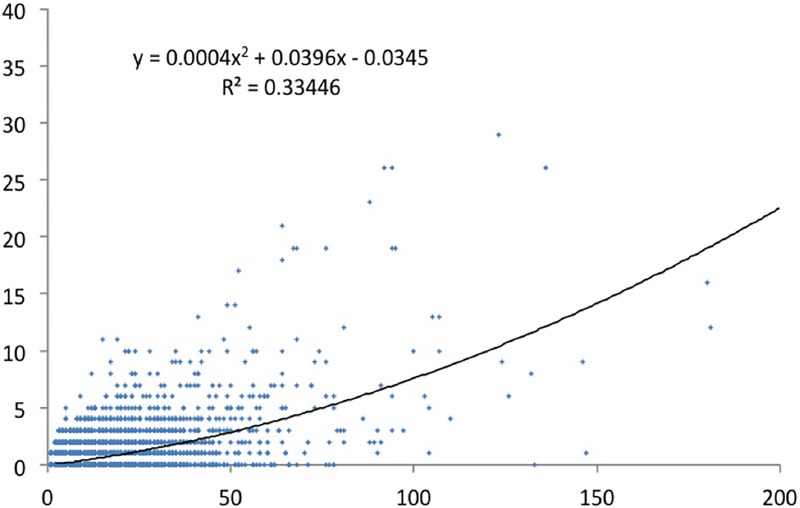
CSS3 cited papers by total number of papers.

**Fig 7:**
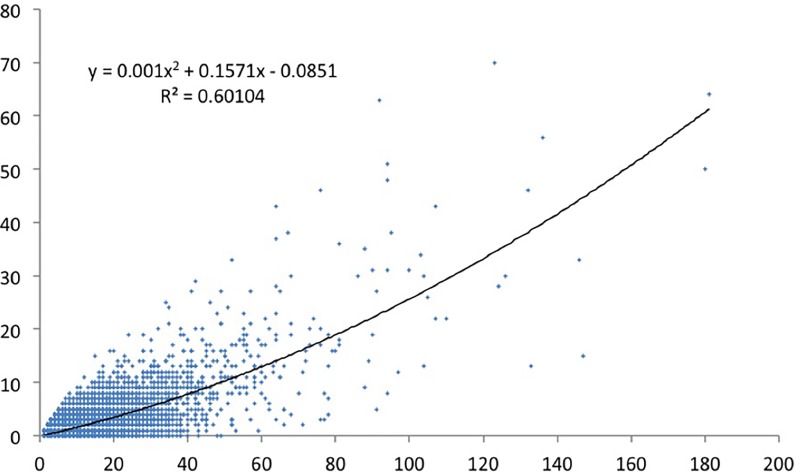
Top 10% cited papers by total number of papers.

**Fig 8:**
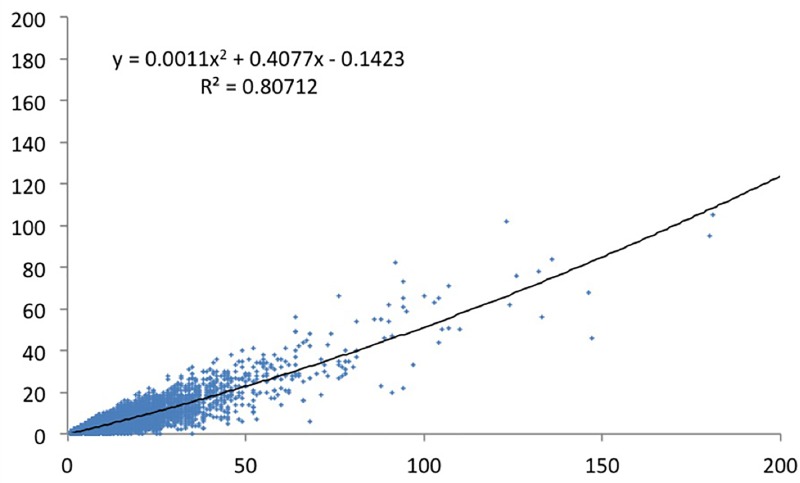
CSS1 cited papers by total number of papers.

We have done a regression analysis with high-cited papers as dependent variable, and productivity as independent variable. We did the analysis for the various definitions of top cited papers. For papers in the top 1% cited papers (Fig 5) the correlation is about 0.5. For the CSS3 (Fig 6), the top 10 cited papers (Fig 7), and the CSS1 classes (Fig 8), the correlations are 0.58, 0.78 and 0.88 respectively. These correlations are fairly high.

The lower the citation threshold the higher the correlation. Why this is the case needs further investigation. One may conclude that the higher the level of citations, the more randomness in the data. However, a probably more obvious explanation is that the small number of high productive researchers with many top papers always have co-authors to these high cited papers who themselves are not highly productive, and may be in the science system only for a few years. This could be PhD students, temporary research staff, or post docs who e.g. have not pursued an academic career and stopped publishing. In that sense one also expects top cited authors in the lower productivity segments, reducing the explained variance. To control for this effect, one may include only PIs in the analysis, something we will address in a subsequent study.

Why would this effect be stronger for the higher citation threshold? This is related to the distribution of productivity. As generally is the case in bibliometric distributions [29], also in our population a small share of all authors produces a large share of the papers and even a larger share of the highly cited papers. For example, the 6.3% most productive researchers in our population (this is everybody with more than eleven publications in four years) are responsible for 37% of all papers and for 53% of the top 1% cited papers. And some 93% of the authors in our sample have no CSS3 top cited paper in the period under consideration,also these figures support the hypothesis that quantity makes a difference. So the higher the threshold, the larger the number of authors that have zero top cited papers also those that have top cited papers in the less selective sets (CSS1, top 10%). The latter authors now score zero and ‘move’ to the X-axis. This increases the dispersion of the data points–and consequently the correlation decreases.

This effect is also stronger if there are more co-authors per paper. This is easily understood, as the more co-authors a top paper has, the more not productive co-authors exist that do have co-authored top-cited papersWe may conclude that co-authoring equalizes and is not beneficial to the small elite. Depending on the field, between 92% and 98% of the researchers does not have a single paper in the CSS3 class in the four years period under consideration. The number of co-authors differs between fields, and indeed this is related to the steepness of the curves in Fig 2. The steeper the curve, the less authors in the lower productivity groups have top cited papers, and one would expect this more in fields with less co-authors per paper. As Table 2 suggests, there is indeed negative relation between the average (and the median) number of co-authors, and the steepness of the curve in Fig 2.

**Table 2:** Co-authors by field.

Area	Relation papers and top papers*	mean	median
Chemistry, Physics, Engineering	1	15.2	4
Life Sciences & Medical Science	2	8.1	5
Agriculture & Food Science	3	6.6	5
Biology, Environmental Science & Geography	4	5.4	4
Psychology & Education	5	4.8	3

* Rank order of steepness of the curves in Fig 2 (right Y-axis only)

### (iii) The effect of fractional counted productivity on the number of high-cited papers?

**Fig 9:**
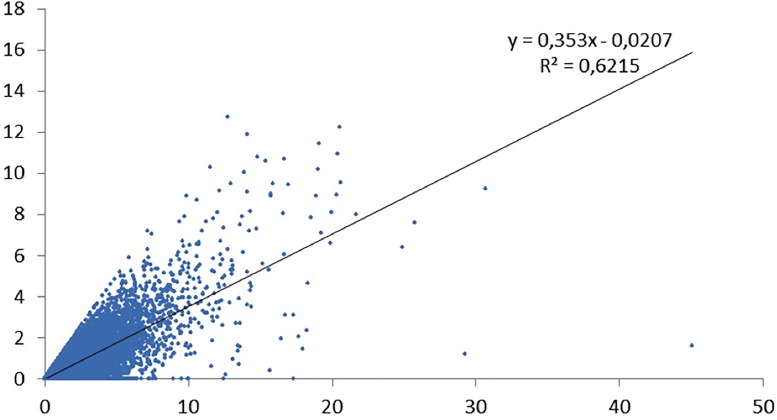
CSS1 cited papers by total number of fractionally counted papers.

**Fig 10:**
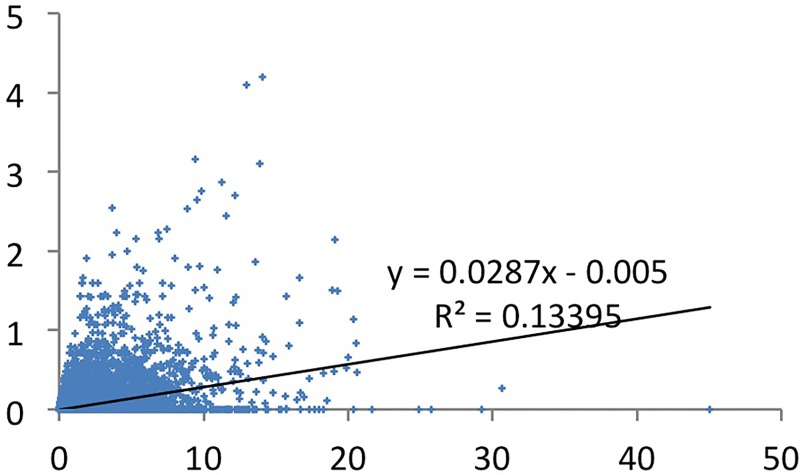
CSS3 cited papers by total number of fractionally counted papers.

We did the above type of analysis also using fractional counting of productivity. The patterns are the same, with correlations of .37 and .79 (Figs 9 and 10). But the correlations are about .10 to .20 lower than in the full counted model. Also here, the co-author phenomenon may influence the result: if the highest cited papers have more co-authors than the other papers, fractionalization reduces the scores of the more productive researchers more, and lowers the correlation coefficient. This is indeed the case, the 6.3% most productive authors have 53% of the full counted PPtop 1% cited papers, but they have 46.8% of the fractional counted PPtop1% cited papers. In any case, the most productive researchers are also here decisive.

### (iv) The effect of field adjusted production counting?

**Fig 11:**
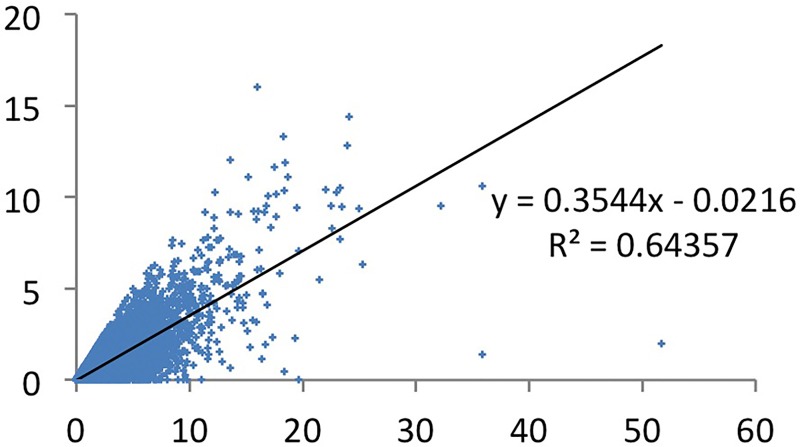
Fractionalized CSS1 by field adjusted production (FAP—all areas of science).

As discussed above, productivity figures differ between fields, and it may therefore be useful to normalize productivity data to field specific averages. The relation between the number of CSS1 papers and the total field adjusted output is strong: the correlation is high with r = 0.80 (Fig 11), and not much smaller than in the above where we did not use the field adjusted production (0.90, see Fig 8). These results suggest that the more papers someone publishes, the higher the number fairly good papers cited above the threshold of CSS1.

**Fig 12:**
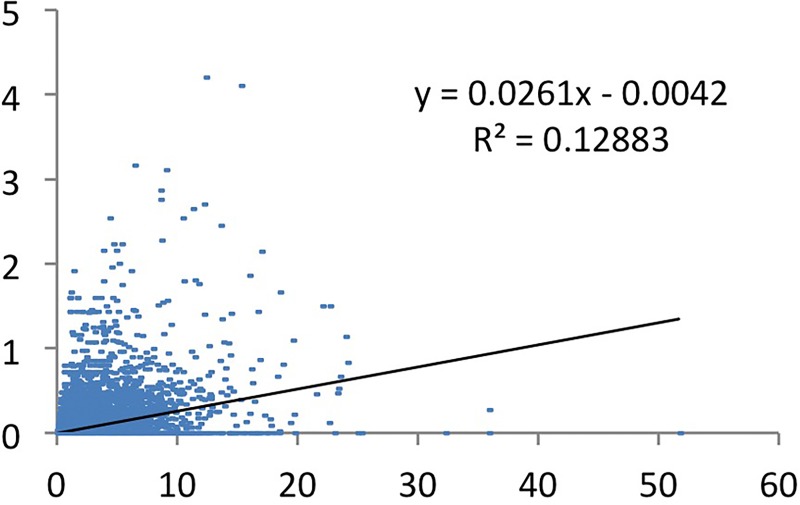
Fractionalized CSS3 by field adjusted production (FAP).

We do the same for CCS3 papers as dependent variable, the much narrower defined top, and field-adjusted productivity as independent variable. If we only include natural and health/life sciences, the correlation is considerable (r = 0.58), and about equally large as without applying FAP (Fig 6). However, if we include all fields in the FAP, the correlation lowers to 0.37 (Fig 12). This may be due to the fact that in some of the other fields the highest productivity levels are almost empty and that in some of those fields, the most productive researchers have a lower share (not a lower number) in the high impact paper class. As discussed above, this may be because they serve other audiences than only direct peers.

## Conclusions

As the above results show, there is not only a strong correlation between productivity (number of papers) and impact (number of citations), that also holds for the production of high impact papers: the more papers, the more high impact papers. More specifically, for most fields there are constant or increasing marginal returns. In that sense, increased productivity of the research system is not a perverse effect of output oriented evaluation systems, but a positive development. It strongly increases the occurrence of breakthroughs and important inventions [16], as would be expected from a theoretical perspective on scientific creativity [13]. Also, we find that other recent work points in the same direction [18; 20]. The lively discussion [e.g. [9; 10] that there is a risk of confusing quality with quantity therefore lacks empirical support. As we deployed a series of methods, with results all pointing in the same direction, the findings are not an artefact of the selected method. The increasing popular policy that allows researchers to hand in only their five or so best publications seems in the light of these results counterproductive, as it disadvantages the most productive and best researchers.

The analysis also gives an indication of the output levels that one may strive at when selecting researchers for grants or jobs. To produce high impact papers, certain output levels seem to be required–of course at the same time dependent on which field is under study.

Future work in this research line will cover various extensions: Firstly, we plan to extend the analysis to some other countries, which of course requires large-scale disambiguation of author names. Secondly, we will in a next version control for number of co-authors, and for gender [30]. The former relates to the discussion about team size and excellence, the latter to the ongoing debate on gender bias and gendered differences in productivity. Thirdly, the aim is to concentrate on principle investigators, and remove the incidental co-authors with low numbers of publications, as they may seem to be high impact authors at the lower side of the performance distribution. This all should lead to a better insight in the relation between productivity and impact in the science system.

## Supporting Information

S1 File Confidence intervals (95%) for the mean number / share of CSS3 papers per field and by productivity class. (PDF)
