# Supplementary material for: Quantity and/or Quality? The Importance of Publishing Many Papers
Source: PLoS One. 2016 Nov 21;11(11):e0166149. doi: 10.1371/journal.pone.0166149 (PMC5117611; doi:10.1371/journal.pone.0166149)
Supplement: S1 File — (PDF) [file pone.0166149.s001.pdf]

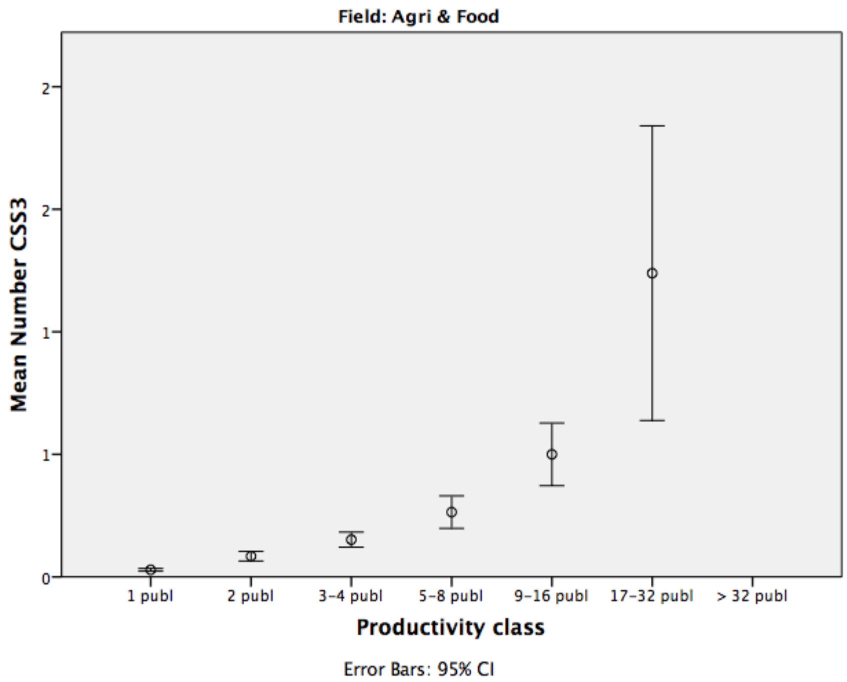

S1 Fig 1. Agriculture and Food Science: 95% confidence intervals for the mean number of CSS3 papers by productivity class

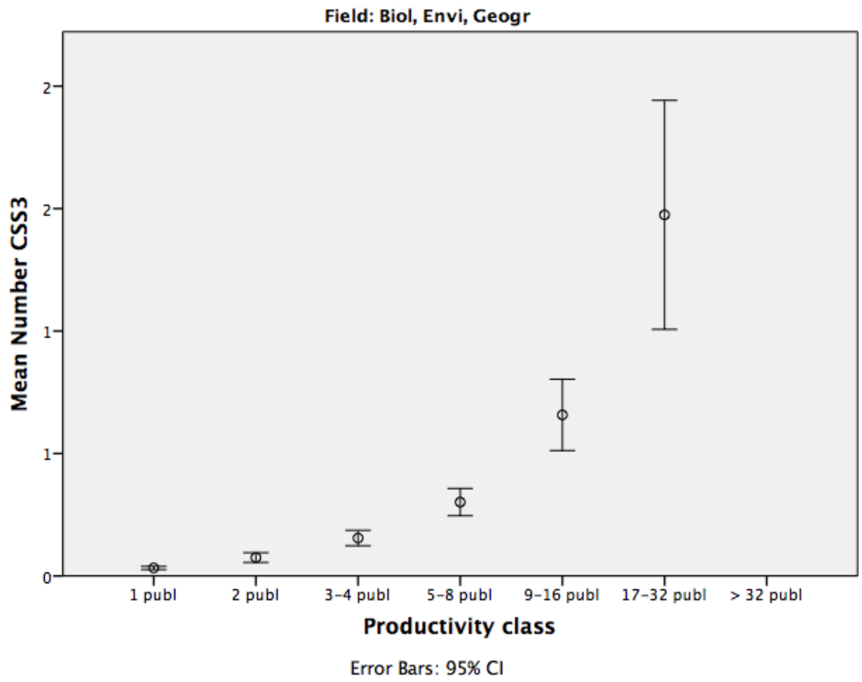

S1 Fig 2 Biology, Environment and Geography: 95% confidence intervals for the mean number of CSS3 papers by productivity class

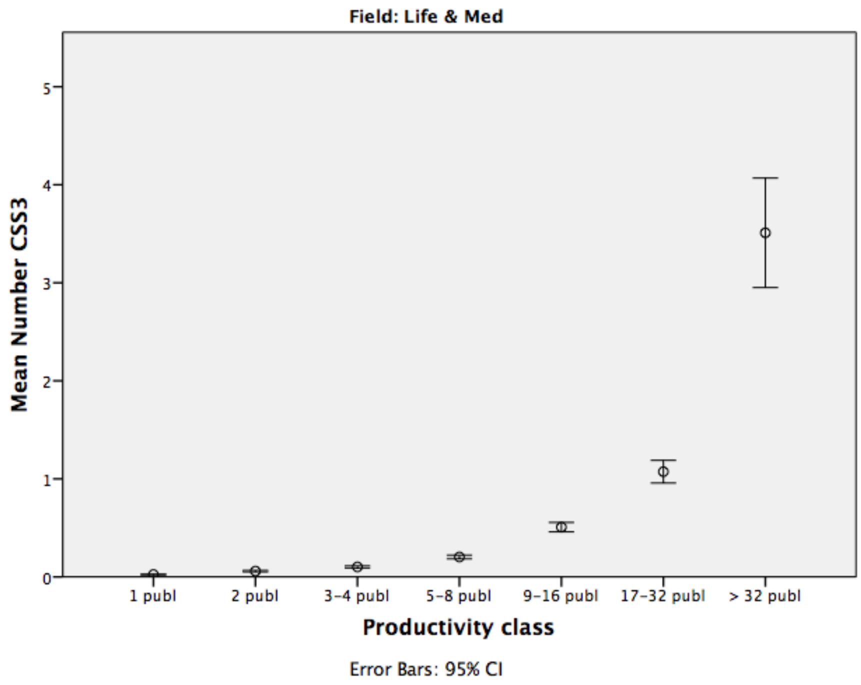

S1 Fig 3. Life Science and Medicine: 95% confidence intervals for the mean number of CSS3 papers by productivity class

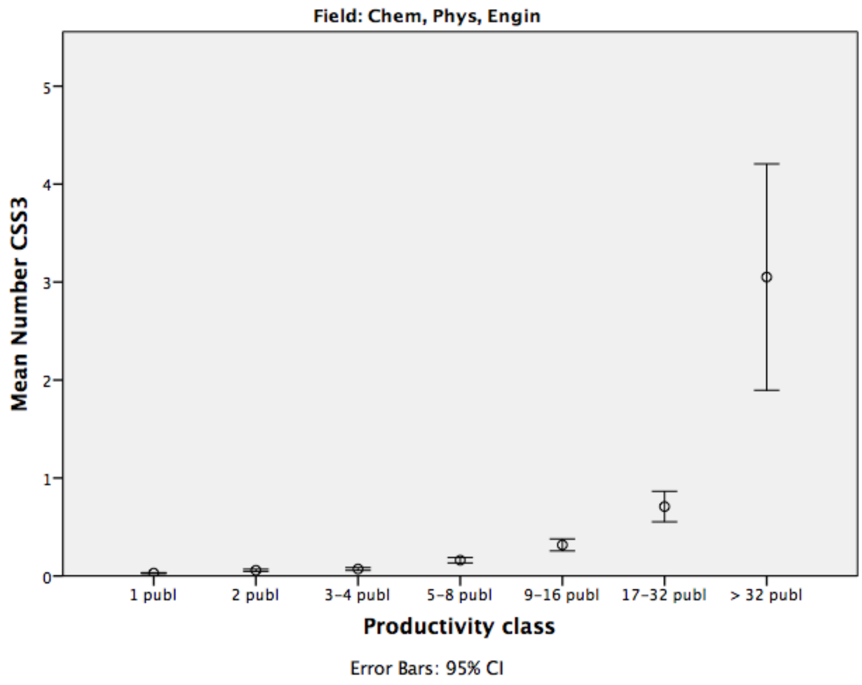

S1 Fig 4. Chemistry, Physics and Engineering: 95% confidence intervals for the mean number of CSS3 papers by productivity class

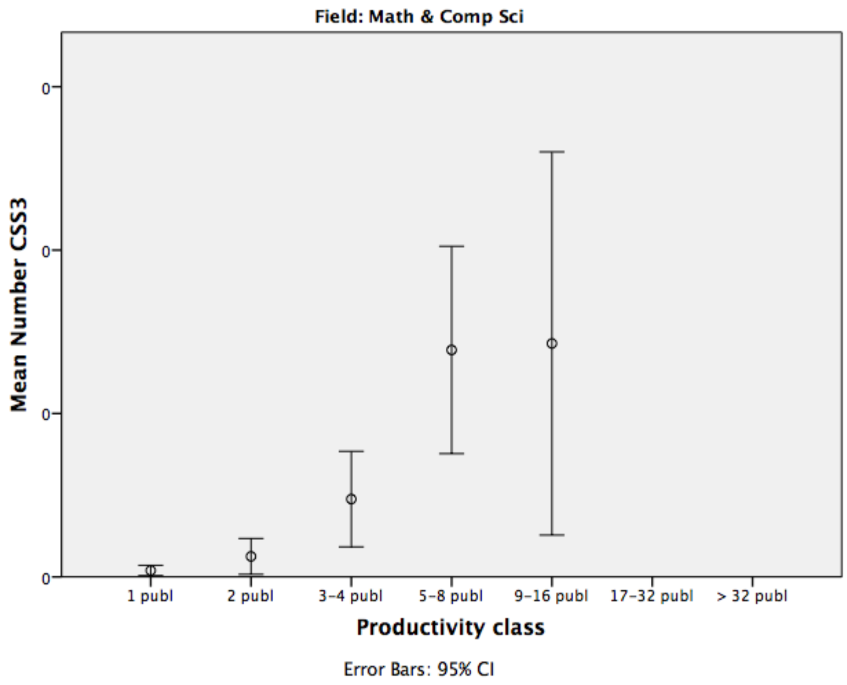

S1 Fig 5. Mathematics and Computer Science: 95% confidence intervals for the mean number of CSS3 papers by productivity class

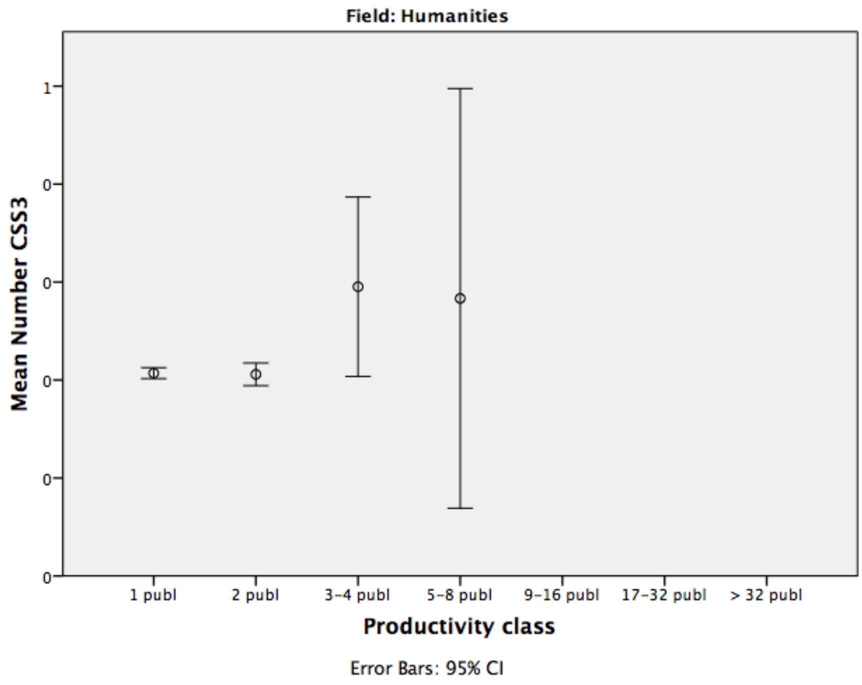

S1 Fig 6. Humanities: 95% confidence intervals for the mean number of CSS3 papers by productivity class

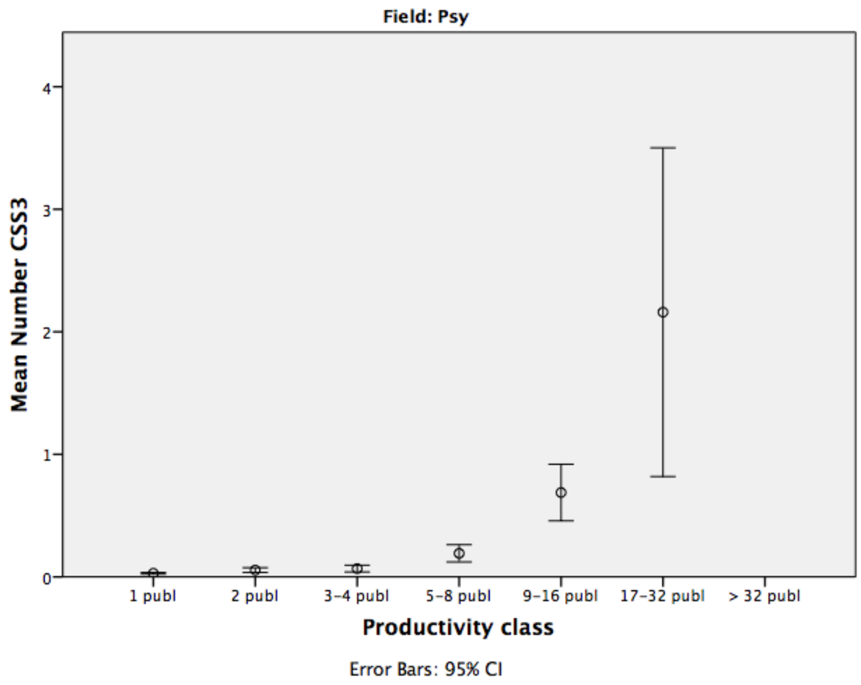

S1 Fig 7. Psychology and Education: 95% confidence intervals for the mean number of CSS3 papers by productivity class

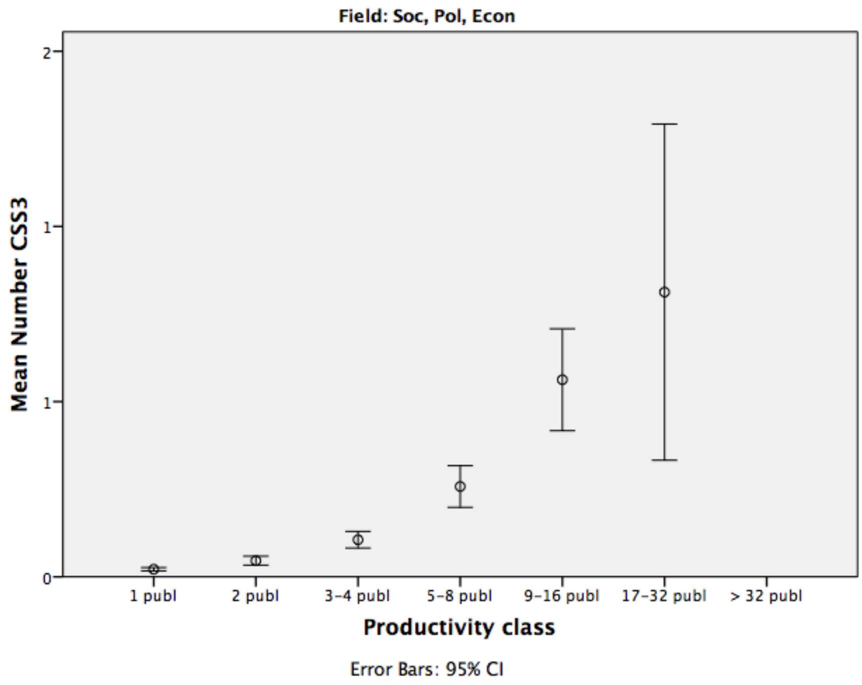

S1 Fig 8. Sociology, Economics, Political Science: 95% confidence intervals for the mean number of CSS3 papers by productivity class

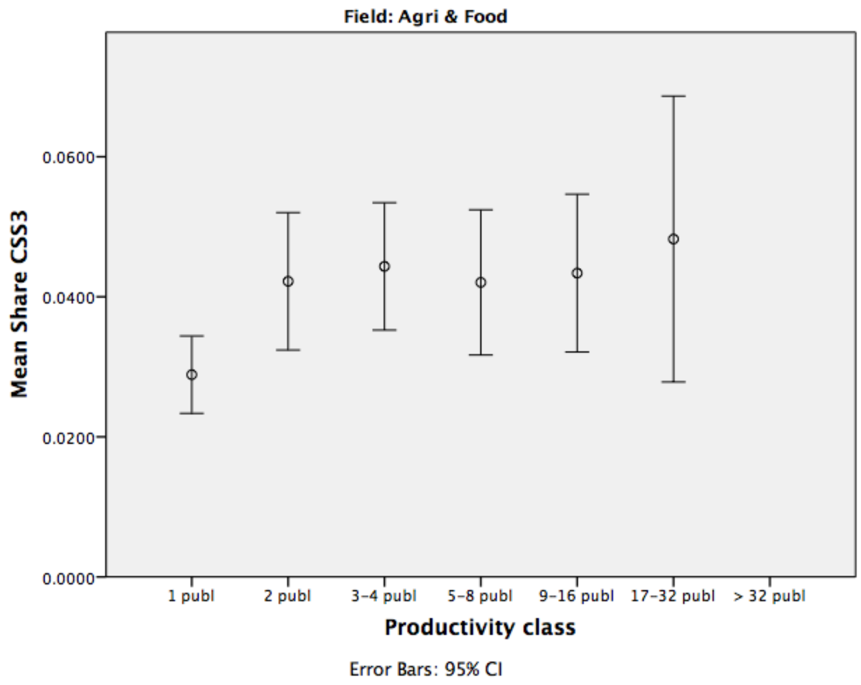

S1 Fig 9. Agriculture and Food Science: 95% confidence intervals for the mean share of CSS3 papers by productivity class

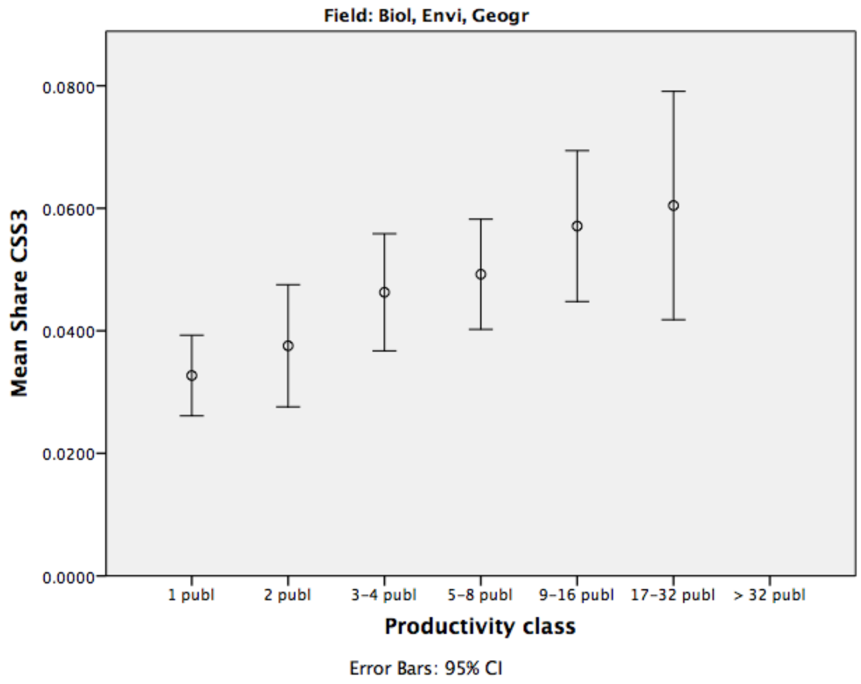

S1 Fig 10. Biology, Environment, Geography: 95% confidence intervals for the mean share of CSS3 papers by productivity class

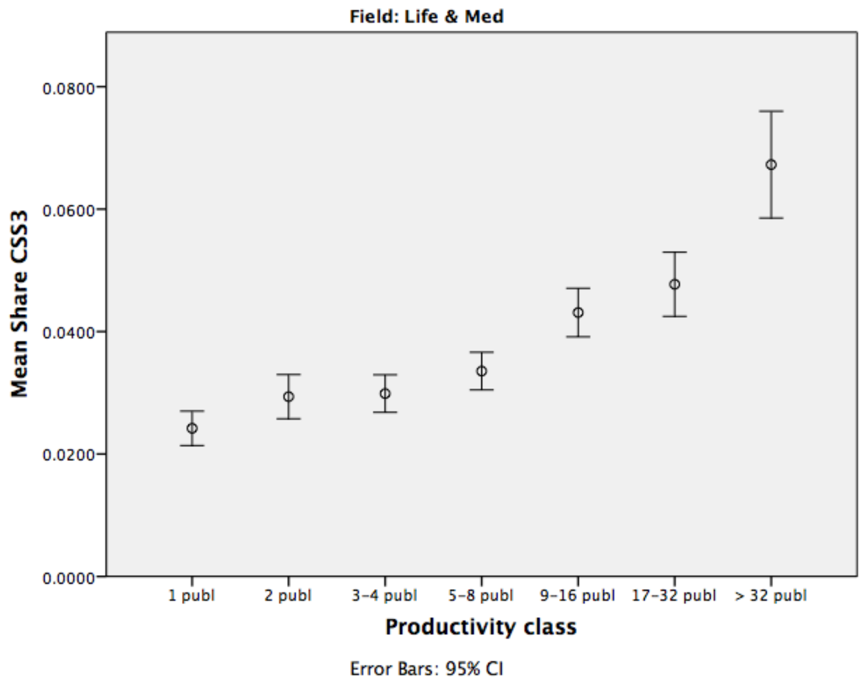

S1 Fig 11. Life Science and Medicine: 95% confidence intervals for the mean share of CSS3 papers by productivity class

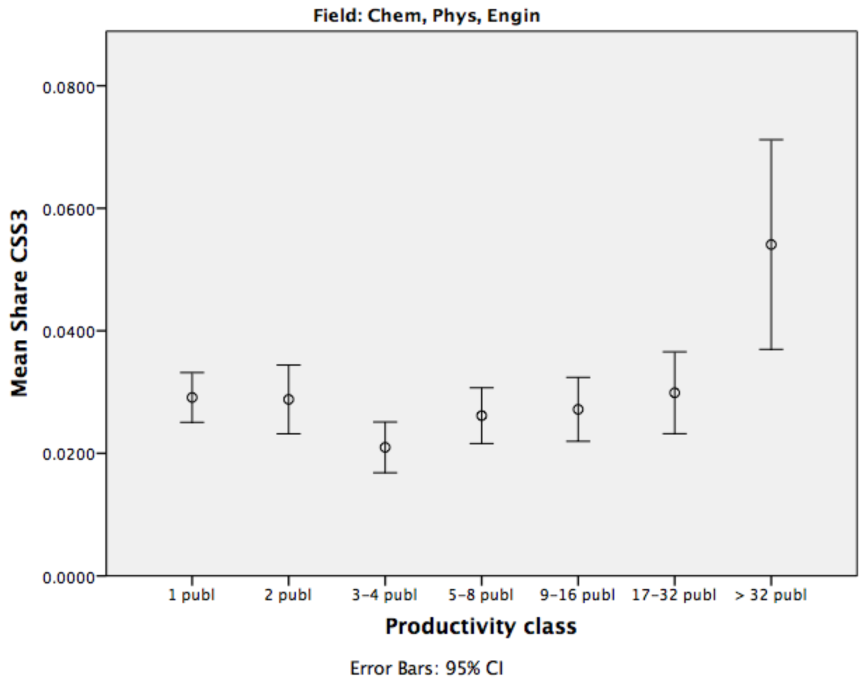

S1 Fig 12. Chemistry, Physics, Engineering: 95% confidence intervals for the mean share of CSS3 papers by productivity class

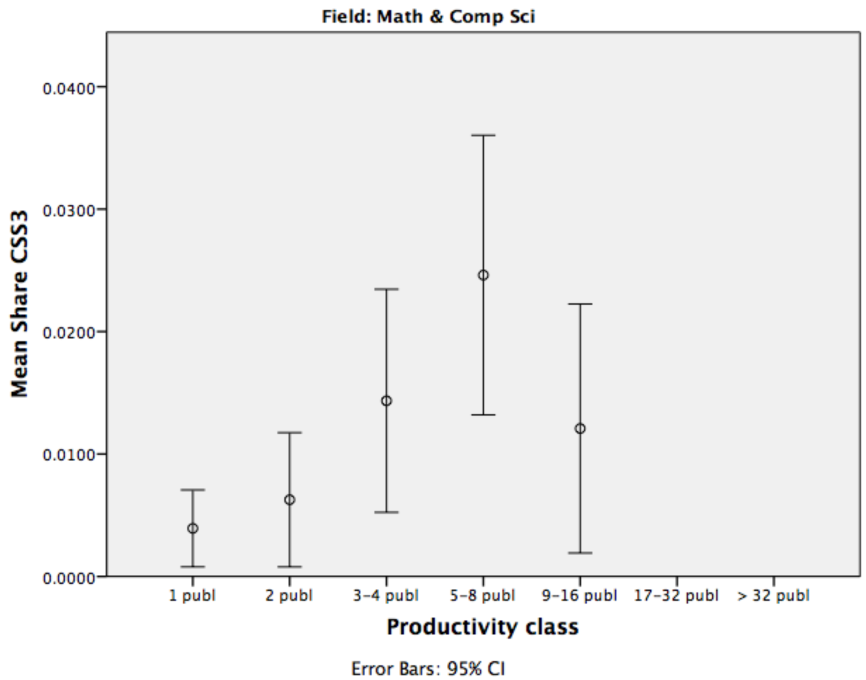

S1 Fig 13. Mathematics and Computer Science: 95% confidence intervals for the mean share of CSS3 papers by productivity class

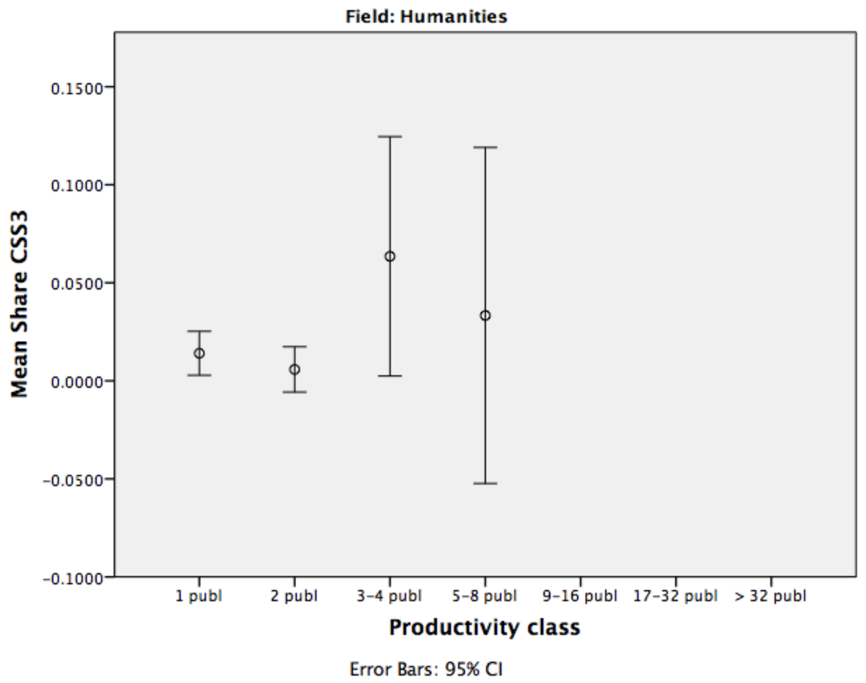

S1 Fig 14. Humanities: 95% confidence intervals for the mean share of CSS3 papers by productivity class

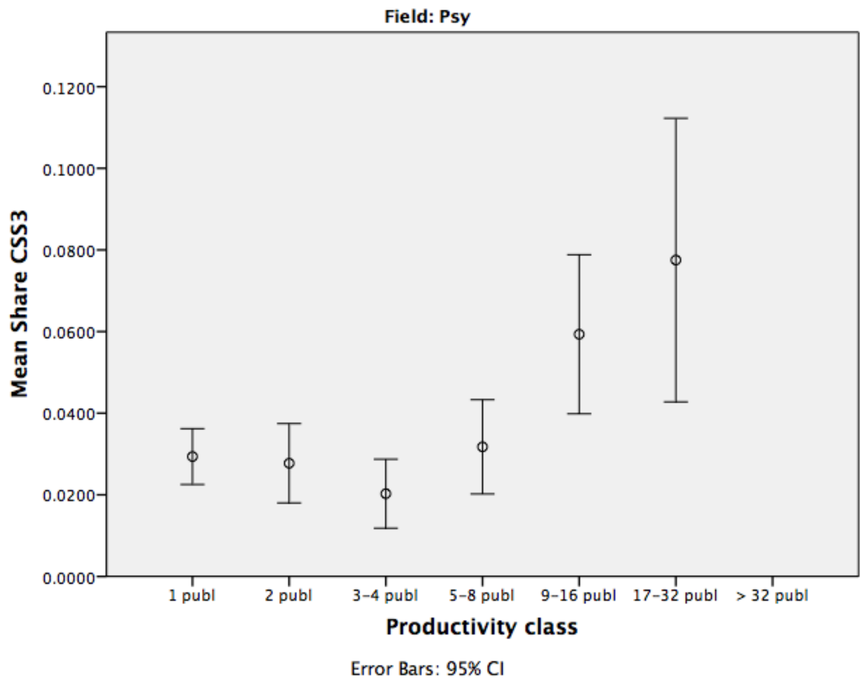

S1 Fig 15. Psychology and Education: 95% confidence intervals for the mean share of CSS3 papers by productivity class

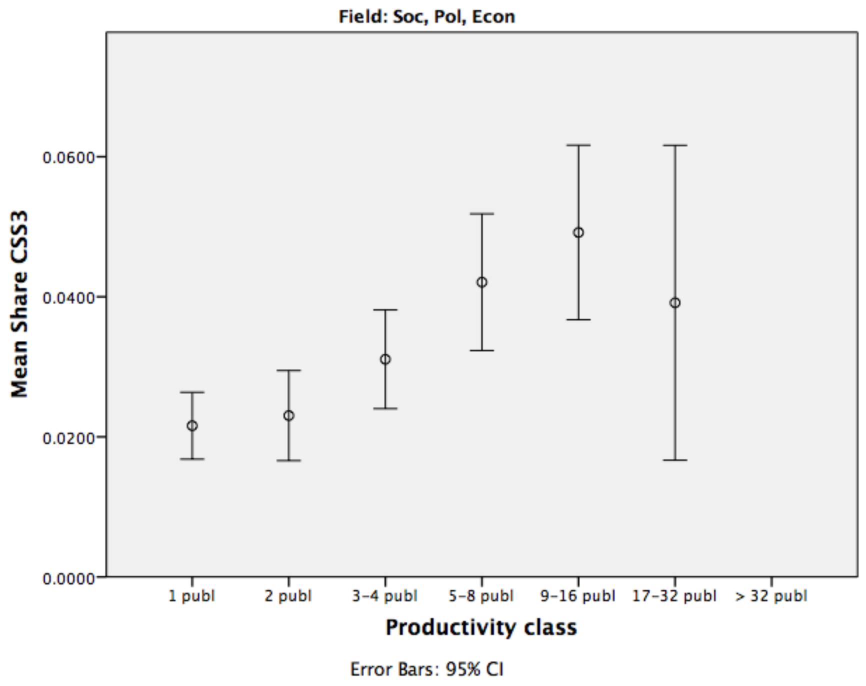

S1 Fig 16. Sociology, Economics, Political Science: 95% confidence intervals for the mean share of CSS3 papers by productivity class
